# Anti-Inflammatory Effects of *Aspalathus linearis* and *Cyclopia* spp. Extracts in a UVB/Keratinocyte (HaCaT) Model Utilising Interleukin-1α Accumulation as Biomarker

**DOI:** 10.3390/molecules21101323

**Published:** 2016-10-02

**Authors:** Tandeka Magcwebeba, Pieter Swart, Sonja Swanevelder, Elizabeth Joubert, Wentzel Gelderblom

**Affiliations:** 1 Department of Biochemistry, Stellenbosch University, Private Bag X1, Matieland (Stellenbosch) 7602, South Africa; tmagcwebeba@sun.ac.za (T.M.); pswart@sun.ac.za (P.S.); 2 Biostatistics Unit, South African Medical Research Council, P.O. Box 19070, Tygerberg 7505, South Africa; sonja.swanevelder@mrc.ac.za; 3 Post-Harvest and Wine Technology Division, Agricultural Research Council (Infruitec-Nietvoorbij), Private Bag X5026, Stellenbosch 7599, South Africa; joubertL@arc.agric.za; 4 Department of Food Science, Stellenbosch University, Private Bag X1, Matieland (Stellenbosch) 7602, South Africa; 5 Institute of Biomedical and Microbial Biotechnology, Cape Peninsula University of Technology, P.O. Box 1906, Bellville 7535, South Africa

**Keywords:** UVB irradiation, herbal tea polyphenols, dihydrochalcones, xanthones, flavanones, antioxidants, anti-inflammatory effects

## Abstract

Ultraviolet B (UVB) radiation is one of the major predisposing risk factors of skin cancer. The anticancer and photoprotective effects of unoxidized rooibos (*Aspalathus linearis*) and honeybush (*Cyclopia*) herbal teas, containing high levels of dihydrochalones and xanthones, respectively, have been demonstrated in skin cancer models in vivo. In the current study, the anti-inflammatory effects of methanol and aqueous extracts of these herbal teas were investigated in a UVB/HaCaT keratinocyte model with intracellular interleukin-1α (icIL-1α) accumulation as a biomarker. Extracts of green tea (*Camellia sinensis*) served as benchmark. Both extracts of green tea and rooibos, as well as the aqueous extract of *C. intermedia*, enhanced UVB-induced inhibition of cell viability, proliferation and induction of apoptosis, facilitating the removal of icIL-1α. The underlying mechanisms may involve mitochondrial dysfunction exhibiting pro-oxidant responses via polyphenol-iron interactions. The methanol extracts of honeybush, however, protected against UVB-induced reduction of cell growth parameters, presumably via antioxidant mechanisms that prevented the removal of highly inflamed icIL-1α-containing keratinocytes via apoptosis. The dual antioxidant and/or pro-oxidant role of the polyphenolic herbal tea constituents should be considered in developing preventive strategies against UVB-induced skin carcinogenesis. The indirect removal of UVB damaged keratinocytes by herbal tea extracts via apoptosis may find application in the prevention of photo-induced inflammation.

## 1. Introduction

Ultraviolet B (UVB) radiation (280–315 nm) is regarded as one of the major predisposing risk factors of skin cancer as it is known to induce DNA damage, oxidative stress, inflammation and cell cycle dysregulation, pathological events that can lead to malignant transformation in cells [1,2,3]. Keratinocytes are the primary targets of UVB-induced toxic effects [4,5], as a result the most commonly occurring skin cancers develop in the epidermal layer through a multistage process involving initiation, promotion and progression [6,7,8]. In the complex process of photocarcinogenesis, two distinct pathways regulating DNA damage repair and inflammation have been identified [9,10,11]. The one pathway occurs upon DNA damage which involves cell cycle arrest, DNA misrepair genetic mutations, cell cycle dysregulation and apoptosis in cells. In the other pathway, UVB activates the production of various pro-inflammatory mediators including the primary (TNF-α and interleukin-1 (IL-1α and β)) and secondary (IL-6, IL-8, IL-10 and IL-4) cytokines that mediate inflammation and immunosuppresion [9,12]. A key signalling molecule responsible for the early inflammatory events in skin is IL-1α, a primary cytokine that is constitutively produced in keratinocytes as a biologically active precursor molecule that remains in the cytosol [13,14,15]. Upon cell activation by inflammatory stimuli, cytosolic IL-1α translocates to the nucleus where it activates the production of pro-inflammatory cytokines and growth factors that are involved in the recruitment of immune cells producing reactive oxygen species (ROS) and cell proliferation [16,17].

In damaged cells, IL-1α is passively released into the extracellular environment where it mediates the inflammatory process via IL-1R-dependent mechanisms. The necrotic release is associated with epidermal hyperplasia and malignant transformation in skin [18,19,20]. Due to its role in inflammation and cancer development, IL-1α has been used as a biomarker in various in vivo and in vitro models to evaluate the progression of the inflammatory process during cancer development [20,21,22]. However, as the necrotic release of IL-1α is associated with an exacerbated inflammatory response, it has been suggested that extracellular IL-1α signalling may not be a suitable model for chemoprevention studies and mechanisms, focusing on the inactivation of intracellular IL-1α (icIL-1α) via apoptosis, have thus been suggested [23]. Consequently, an in vitro UVB irradiation cell model was developed in which induction of icIL-1α could be inhibited either by transcription modulation or augmentation of apoptosis [24]. Since, in vitro models, utilising primary or cultured keratinocytes have served as useful tools in mechanistic and drug development studies of inflammation [25,26,27,28], the UVB/HaCaT model was also recommended as a screening tool that could be used to determine the anti-inflammatory and chemopreventive efficacy of novel compounds in the inflammatory process.

Naturally occurring antioxidants such as polyphenolic plant constituents have been the focus of research studies concerned with the development of novel anti-inflammatory and chemopreventive agents in skin [29,30,31,32]. One of the most extensively studied natural agents in skin is green and black tea (*Camellia sinensis*) and their polyphenolic constituents, which have been shown to prevent inflammation by inhibiting the synthesis of IL-1α in mouse skin. The latter is suggested to be one of the underlying mechanisms involved in the anti-tumour activity of both green and black tea extracts [33,34].

Rooibos (*Aspalathus linearis*) and honeybush (*Cyclopia*) species are South African herbal teas that have traditionally been used in the treatment of skin disorders and in skin care products due to their antioxidant, anti-inflammatory, immunomodulatory, anti-proliferative and anticancer activities [35,36,37]. These biological properties, partly attributed to the dihydrochalcones of rooibos and the xanthones and flavanones of honeybush, have also been associated with the prevention of various chronic illnesses including cancer. The anti-carcinogenic properties of rooibos and honeybush (*C. intermedia*) extracts have been demonstrated in mouse skin carcinogenesis models [38,39]. Modulation of inflammation by the polyphenolic constituents has been proposed as a key mode of action underlying the protective activity of the extracts. The anti-inflammatory properties of rooibos and honeybush against UVB and PMA-induced COX-2 expression and oedema have been demonstrated and implicated in the anti-tumour activity of the herbal tea extracts in skin [39,40]. However, the modulatory activity of the herbal teas against primary cytokines involved in the early stages of UVB-induced inflammation as well as the effect on cell growth parameters still needs to be elucidated. 

The aim of the current study was therefore to determine the effect of methanol and aqueous extracts of rooibos and two honeybush species against UVB-induced icIL-1α accumulation in keratinocytes in relation to various cell growth indices. Green tea served as a benchmark. The extracts used in the current study were the same used previously, in the evaluation of the potential role of polyphenols in the modulation of skin cell viability by *Aspalathus linearis* and *Cyclopia* spp in vitro [24]. Depending on the extract type, the herbal teas showed differential effects in the reduction of icIL-1α in UVB-exposed HaCaT keratinocytes and this was closely related to the reduction in cell viability and induction of apoptosis. 

## 2. Results

### 2.1. Comparative Effects of Extracts in UVB Irradiated and Non-Irradiated HaCaTs

#### 2.1.1. Cell Viability (Table 1)

Green tea and rooibos were the most active in reducing cell viability based on cellular ATP content when compared to the two honeybush species. The methanol extract of green tea was more active than its aqueous extract both in the absence and presence of UVB irradiation. The rooibos methanol extract was significantly more active (*p* < 0.05) in the presence of UVB, while aqueous extract exhibited a similar activity between non-irradiated and UVB irradiated cells. The rooibos methanol extract was more active than its aqueous extract in the presence of UVB irradiation. In contrast, aqueous extracts of *C. intermedia* and *C. subternata* were more effective (*p* < 0.05) in reducing cell viability than their methanol extracts in non-irradiated and UVB irradiated cells. In the UVB irradiated cells, the honeybush methanol extracts not only exhibited a far weaker response, but no IC_50_ values could be determined against cell viability at the levels tested. The aqueous extract of *C. intermedia* exhibited the highest activity while both honeybush species exhibited a similar activity in UVB-irradiated and non-irradiated cells. 

#### 2.1.2. Cell Proliferation (Table 1)

The green tea and rooibos extracts were the most effective in the inhibition of cell proliferation either in the absence or presence of UVB irradiation with the extract type having no significant effect (*p* ≥ 0.05). Both *Cyclopia* extracts exhibited a similar inhibition of cell proliferation in non-irradiated cells. No IC_50_ values could be determined for their methanol extracts following UVB irradiation at the levels tested. The aqueous extracts of *C. intermedia* and *C. subternata* exhibited a similar inhibitory effect, although lower (*p* < 0.05) IC_50_ values were noticed in the absence of UVB irradiation. In the presence of UVB irradiation, *C. intermedia* exhibited a significant (*p* < 0.05) higher activity, i.e., lower IC_50_ value.

#### 2.1.3. Modulation of UVB-Induced Apoptosis in Relation to Cell Viability (Table 2)

Varying doses of the different extracts were used to investigate the modulation of apoptosis based on the variable effects noticed on cell viability and cell proliferative indices. Dose levels for green tea and rooibos methanol extracts were selected based on IC_50_ concentrations of the tea/herbal tea against cell viability and cell proliferation (Table 1). The green tea and rooibos methanol extracts exhibited the highest activity in enhancing UVB-induced apoptosis in a dose-dependent manner, as evidenced by the fold increase. Their aqueous extracts also exhibited a similar effect although at three- to four-fold higher concentrations. The green tea aqueous extract tended to be more effective than rooibos as evidenced by a higher activity at a lower dose level. The induction of apoptosis by green tea and rooibos extracts was closely related to a reduction of cell viability.

As the honeybush aqueous extracts were less effective against cell viability higher concentrations, deduced from the highest IC_50_ levels against cell viability, were utilised. The same argument was used when considering their methanol extracts, although the latter lacked an IC_50_ against cell viability (Table 1). Contrary to green tea and rooibos, the methanol extracts of *C. intermedia* and *C. subternata* reduced (*p* < 0.05) caspase-3 activity in a dose-dependent manner with the highest concentrations exhibiting the strongest effect. This reduction in caspase-3 activity was either comparable to the negative control in the case of *C. intermedia* or similar to the positive control for *C. subternata.* The reduction of apoptotic activity by the honeybush species was accompanied by a relatively weaker inhibition of cell viability when compared to green tea and rooibos even at approximately three- to four-fold higher concentrations. The aqueous extracts, similar to green tea and rooibos, increased caspase-3 activity with a resultant decrease in cell viability at the highest concentration. 

#### 2.1.4. Modulation of UVB-Induced icIL-1α Accumulation

**Figure 1:**
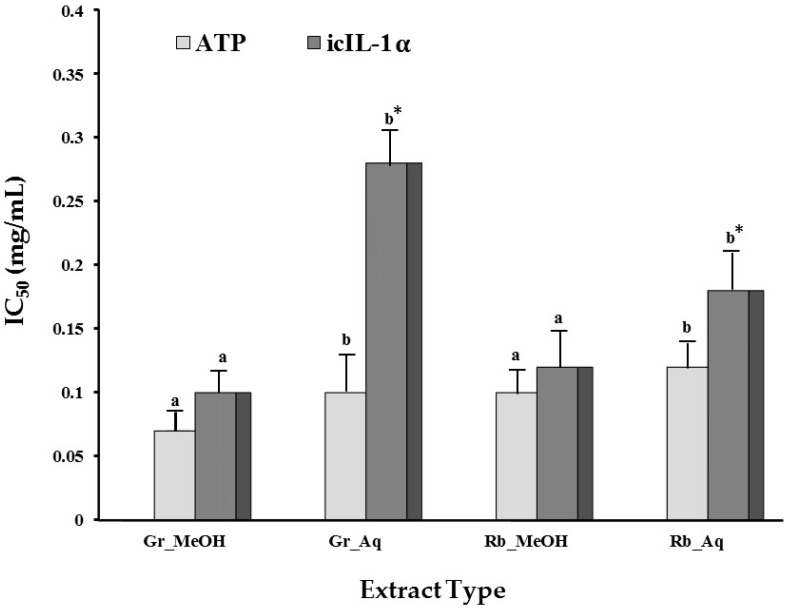
Modulation of cell viability (ATP content) and icIL-1α accumulation by green tea and rooibos tea in relation to their respective IC_50_ concentrations. Values are means of duplicate determinations with five repetitions each. Different letters implies significant (*p* < 0.05) differences between the means of the methanol and aqueous extracts within the selected parameter, respectively. Means between * Gr_MeOH and Rb_MeOH (IL-1α) and # Gr_MeOH and RB_MeOH (ATP) differ significantly (*p* < 0.05). *Abbreviations*: Gr—green tea; Rb—rooibos; Aq—water; MeOH—methanol.

The methanol and aqueous extracts of green tea and rooibos inhibited UVB-induced icIL1-α accumulation in a dose-dependent manner (data not shown), while exhibiting no release of extracellular IL-1α (exIL1-α). The latter varies between 2 and 4 pg icIL-1α per mL of the supernatant in the UVB positive control and treatment protocols with the tea/herbal teas with no significant differences (*p* > 0.05) irrespective the treatment regimen (Table 3) When considering the IC_50_ values, the methanol extracts were significantly (*p* < 0.05) more effective in reducing icIL-1α than their aqueous extracts with green tea exhibiting a significant higher activity when compared to rooibos (Figure 1). The IC_50_ value of the aqueous extract of green tea and rooibos were of the same order. A smilar trend is noticed with respect to the reduction of cell viability indicating that the reduction of icIL-1α was related to the decrease in cell viability (ATP content). However, slightly lower extract IC_50_ concentrations were required for the methanol extracts in reducing cell viability when compared to the reduction in icIL-1α accumulation. For the aqueous extract, a significantly (*p* < 0.05) higher IC_50_ concentration was noticed for the reduction of icIL-1α. 

The aqueous extract of *C. intermedia* exhibited a dose-dependent inhibition of icIL-1α which was associated with a decrease in cell viability similar to that obtained for green tea and rooibos (Table 4). As reported for green tea and rooibos the exIL-1α levels were not increased and in some cases significantly (*p* < 0.05) decreased. Interestingly, the aqueous extract of *C. subternata* enhanced icIL-1α production at the two highest concentrations despite a significant reduction in cell viability. In contrast to green tea, rooibos and the aqueous extract of *C. intermedia*, the methanol extracts of both honeybush species increased (*p* < 0.05) icIL-1α mainly at the highest concentration used without significantly affecting cell viability.

### 2.2. Relationships between Cell Viability, Apoptosis, Induction of icIL-1α and Major Polyphenolic Constituents

Considering green tea and rooibos extracts, an inverse (*p* < 0.0001) correlation existed between caspase-3 activity and cell viability (ATP content), as well as between the induction of caspase-3 and the reduction in IL-1α (Table 5). In contrast, a positive (*p* < 0.0001) correlation was noticed between cell viability and reduction in IL-1α. The polyphenol content of the green tea and rooibos extracts at their respective IC_50_ values was calculated to provide some insight into the possible role of phenolic sub-classes and major individual compounds (Table 6). The TP and FLAVA (monomeric flavanols and polymeric tannin-like compounds including the proanthocyanidins) content of green tea associated with the IC_50_ for the decrease in icIL-1α accumulation, was higher, specifically the FLAVA content which as approximately three-fold higher when compared to rooibos. Higher levels were also associated with the aqueous extract in the reduction of icIL-1α when compared to the methanol extract due to an increased IC_50_ concentration of the extract (Table 6). A similar pattern was also noticed for the caffeine (5.75 ± 0.16 (methanol) vs. 11.23 ± 0.08 (aqueous) mg/mL) and individual flavanol constituents associated with the IC_50_ value of icIL-1α accumulation of the aqueous extract. The high level of EGCG associated with the inhibition of icIl-1α by the green tea aqueous extracts is of particular interest. With respect to cell viability, higher TP and FLAVA levels were associated with the IC_50_ of the aqueous extract of green tea while the individual flavanols were variable i.e., no difference existed for EGCG between the IC_50_ of the aqueous and methanol extracts. The caffeine equivalents of the methanol and aqueous extracts, associated with the IC_50_ level against cell viability also do not differ (4.03 ± 0.11 vs. 4.01 ± 0.03 mg/mL).

**Figure 2:**
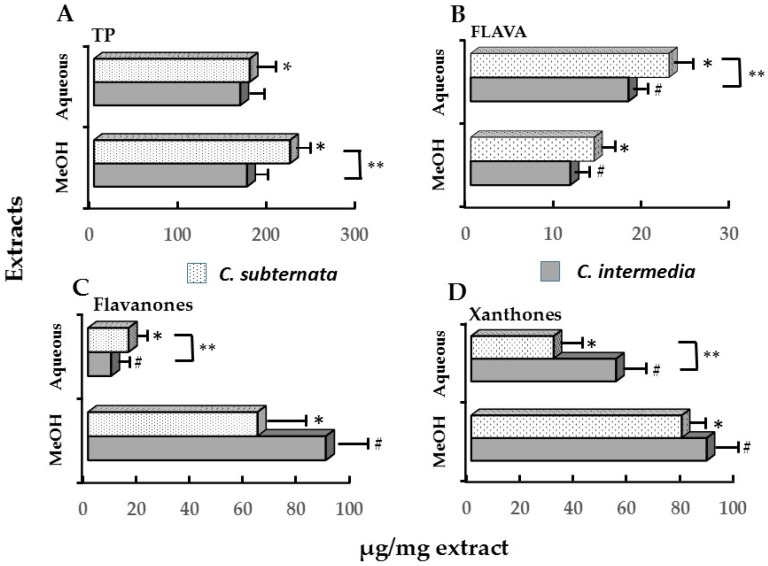
Quantitative levels of: (**A**) TP (total polyphenol); (**B**) FLAVA (flavanol/proanthocyanidin); (**C**) flavanone (hesperidin, eriocitrin and eriodictyol-glycoside); and (**D**) xanthones (mangiferin and isomangiferin) content of the methanol (MeOH) and aqueous (Aq) extracts of *C. subternata* and *C. intermedia.* Values represent means of triplicate determinations. *^,^# Means of methanol and aqueous extracts of a *Cyclopia* species differ significantly (*p* < 0.05). ** Significant (*p* < 0.05) species differences. Data derived from [24].

The TP and FLAVA levels of rooibos extracts associated with the IC50 values the inhibition of icIL-1α were also higher when compared to the IC_50_ values for cell viability. Of interest is that, for rooibos, similar levels of FLAVA, dihydrochalcones, flavones and flavonols were associated with the IC_50_ values for the inhibition of icIL-1α by the methanol and aqueous extracts. However, the levels of these compounds in the methanol extract associated with the IC_50_ value for cell viability were slightly higher when compared to to that of the aqueous extract (Table 6). The aqueous extract of *C. intermedia* exhibited a similar effect (Table 5) to rooibos and green tea as a negative correlation existed between the induction of caspase-3 activity and reduction in icIL-1α content, as well as between caspase-3 activity and cell viability. A positive correlation was also obtained between cell viability and reduction in icIL-1α content. No correlation was noticed for the methanol extracts except for a weak negative correlation between caspase-3 activity and icIL-1α content. The effects of the *C. subternata* extracts were more variable. Similar to *C. intermedia*, a strong inverse relationship was noticed between caspase-3 activity and cell viability for the aqueous extract, but a moderate negative correlation was obtained for its methanol extract. Weak negative correlations also existed between icIL-1 α content and cell viability for both extracts while a moderate positive correlation existed between caspase-3 activity and Il-1α content only for the aqueous extract of *C. subternata*. Only the aqueous extracts of the honeybush species could affect an IC_50_ value for the reduction of cell viability while the methanol extracts lacked an effect on the reduction of icIL-1α accumulation (Table 4). Therefore, the level of individual polyphenolic constituents associated with the IC_50_ values for the reduction of cell viability was only calculated for the aqueous extracts of both honeybush species (Table 7). As a dose-dependent inhibitory response on the accumulation of icIL-1α was obtained for the aqueous extract of *C. intermedia*, an IC_50_ value could also be recorded. A comparison of the phenolic sub-classes between the aqueous and methanol extracts of *C. intermedia* and *C. subternata* is depicted in Figure 2.

The IC_50_ values for the reduction icIL-1α (*C. intermedia*) and cell viability (both species) were above that obtained for green tea and rooibos (Table 4 and Table 7). Higher TP (Figure 2A; only *C. subternata*), flavanone (Figure 2C) and xanthone (Figure 2D) and lower FLAVA levels (Figure 2B) were noticed for the methanol extracts of *C. intermedia* and *C. subternata* when compared to the aqueous extracts. This was associated with a dose-dependent increase in the level of icIL-1α (Table 4). In this regard the methanol extract of *C. subternata*, lacking an IC_50_ for the reduction of icIL-1α, contained significantly (*p* < 0.05) lower TP (Figure 2A), flavanone (Figure 2C), xanthones (Figure 2D) and higher FLAVA (Figure 2B) levels when compared to *C. intermedia*. Of particular interest was the effect noticed for the aqueous extract of *C. subternata* that stimulated icIL-1a accumulation at higher concentrations despite the reduction in cell viability (Table 4). Differences in the polyphenolic sub-classes may be responsible for this differential effect on icIL-1α inhibition between the two species. However, the IC_50_ value of the aqueous extract of *C. subternata* for the reduction in cell viability is associated with similar xanthone and significantly (*p* < 0.05) higher flavanones, flavones, phloretin-3′,5′-di-*C*-glucoside (dihydrochalcone) and, iriflophenone-3-*C*-glucoside (benzophenone) levels when compared to *C. intermedia* (Table 7).

## 3. Discussion

One of the adverse biological effects in skin following UVB radiation involves the increased production and release of the primary cytokine, intracellular IL-1α (icIL-1α) by keratinocytes [41]. The release leads to the subsequent activation of other epidermal and dermal cells resulting in the induction of an inflammatory response and cell proliferation [16,42]. Chronic release, as mediated by different external factors such as exposure to UVB or cancer tumour promoters, is associated with epidermal hyperplasia and skin carcinogenesis [18,25]. Since extracellular IL-1α is associated with an exacerbated inflammatory response and the pathogenesis of disease, mechanisms involving inactivation of icIL-1α via transcription modulation and/or programmed cell death have been identified and recommended as a target in chemoprevention [16,23,43]. UVB radiation is also known to increase the level of ROS by disrupting mitochondrial function causing oxidative DNA damage [4,44].

The protective mechanism against DNA damage involves the induction of cell cycle arrest that allows for DNA repair while irreparable damage triggers apoptosis in a p53 dependent manner [5]. Unrepaired DNA lesions can generate mutations that confer a selective growth advantage to pre-neoplastic cell populations as they become more resistant to apoptotic cell death eventually resulting in the development of cancer. Therefore, a key step proposed during chemoprevention of UVB-induced cell damage is the elimination of transformed cells by apoptotic cell death [45]. 

Naturally occurring plant polyphenols have been targeted for use in cutaneous photo protection as they can modulate UVB-induced inflammatory and apoptotic signalling pathways associated with oxidative stress and DNA damage in skin [30,32,45]. Rooibos and honeybush also possess antioxidant and anti-inflammatory properties that have been implicated in the photoprotective and anti-carcinogenic mechanisms in skin [38,39,40]. The anti-inflammatory activity against cytokine production by quercetin, rutin and luteolin, also present in rooibos, has been shown to involve inhibition of pro-inflammatory cytokine synthesis of via suppression of transcription factors and MAP kinases [31,46]. Mangiferin and hesperidin, major polyphenolic constituents of honeybush, were shown to inhibit the inflammatory response by suppressing cytokines as well as other pro-inflammatory mediators such as adhesion molecules and prostaglandins in vitro and in vivo [47,48,49]. However, the activity of these herbal teas against the adverse effects of UVB irradiation on cell integrity and the induction of primary cytokines still needs to be elucidated. Recently, an in vitro UVB model HaCaT cell model for chemoprevention has been developed for screening the anti-inflammatory efficacy of novel compounds against IL-1α accumulation [43]. The anti-inflammatory activity exhibited against icIL-1α could either involve direct modulation of transcription machinery or pro-apoptotic effects via enhanced caspase-3 activity. Therefore the current study investigated the modulatory activity of methanol and aqueous extracts of rooibos and two selected *Cyclopia* species against icIL-1α accumulation and different cell growth parameters in the UVB/HaCaT keratinocytes chemoprevention in vitro model. The activity of the herbal tea extracts was benchmarked against extracts of green tea, the protective properties of which have been studied extensively in skin [50]. Green tea and rooibos extracts inhibited UVB-induced icIL-1α accumulation without increasing the levels of exIL-1α. The effect on icIL-1α was strongly correlated with reduction in cell viability suggesting that the inhibitory effect on icIL-1α production resulted from a reduction in cell viability and not direct modulation of its transcription machinery as previously demonstrated for dexamethasone and ibuprofen [43]. Since the extracts did not induce the release of icIL-1α, reduction of cell viability could not be associated with necrotic cell death. A strong inverse correlation between icIL-1α inhibition and enhanced UVB-induced caspase-3 activity indicated that the anti-inflammatory activity of the extracts against icIL-1α is indirect and it involves the removal of cells with increased levels of icIL-1α via apoptosis. It is known that, in cells undergoing apoptosis, icIL-1α is retained in the nuclear chromatin fraction thus preventing its release into the extracellular environment [23]. A similar mechanism is therefore proposed in the present study whereby rooibos and green tea extracts control sterile inflammation by promoting nuclear retention of UVB-induced icIL-1α in the keratinocytes via apoptosis.

The pro-apoptotic activity of the green tea extracts in the present study may involve the major compounds, EGCG and the alkaloid, caffeine, since the methanol extract, exhibiting stronger activity in skin cells, was previously shown to contain the highest levels of these compounds [24]. These green tea constituents, specifically EGCG, have been shown to prevent tumour formation by enhancing apoptosis in UVB irradiated mouse skin in vivo [51,52]. In addition, the underlying mechanisms involved in the pro-apoptotic activity of caffeine have also been demonstrated in a HaCaT cell premalignant model known to have UVB typical p53 mutations [53]. Caffeine was shown to specifically targets keratinocytes with unrepaired DNA damage by inhibiting AKT/protein kinase B involved in UVB-induced anti-apoptotic mechanisms. Since the pro-apoptotic activity of rooibos and green tea extracts in the HaCaT cell line was also demonstrated following UVB-irradiation in the present study, these extracts also seem to target cells with unrepaired DNA damage. This is corroborated by the previous study where the HaCaT keratinocytes was also the most sensitive cell line with respect to the reduction in cell viability by rooibos and green tea extracts when compared to normal and cancer skin cells [24]. Although caffeine was significantly higher in the methanol extract of green tea, no appreciable difference in the alkaloid equivalents associated with the IC_50_ level against cell viability was noticed between the methanol and aqueous extracts. Therefore, even though caffeine is likely to play a role in the pro-apoptotic activity of green tea it may not be the main active constituent. In rooibos, the polyphenolic equivalents (as TP and FLAVA), as well as the individual flavonoids associated with IC_50_ values against cell viability, were also similar between the methanol and aqueous extracts. However, when considering apoptosis, the methanol extract, known to have high levels of the monomeric flavonoids and FLAVA constituents, was more active, further emphasising the role of pro-oxidant effects of these polyphenolic compounds in the promotion of UVB-induced apoptosis associated with oxidative stress [24]. Of the rooibos polyphenols very little is known about the anti-inflammatory effects of aspalathin, a dihydrochalcone and major flavonoid present in unoxidized rooibos. Quercetin and rutin have been reported to effect apoptosis in cancer cells including UVB-irradiated HaCaT keratinocytes [54,55]. Iso-orientin and vitexin are also known to effect apoptosis in cancer cells by disrupting the mitochondrial signalling pathways [56,57].

When considering the effect of green tea and rooibos extracts against cell viability and cell proliferation, no significant differences between UVB irradiated and non-irradiated cells were noticed, indicating that these extracts do not offer any protective effect against UVB in cells. Since UVB is known to induce oxidative stress in cells [4], this suggests that the extracts do not provide protection against ROS-induced cellular damage. This implies a synergistic pro-oxidative interaction between UVB and the polyphenolic constituent in the green tea and rooibos extracts. The underlying mechanism is likely to involve polyphenol/iron interactions and defects of the mitochondrial respiratory chain complexes that are related to iron depletion [24,58,59]. In vitro studies have indicated that complex II defects in the mitochondrial electron transport chain can result in decline of ATP levels, cell cycle arrest and oxidative stress [60,61]. Complex II defects have also been shown to mediate UVA-induced ROS production in HaCaT epidermal keratinocytes [44]. Therefore, it is likely that reduction of cell viability and cell proliferation exhibited by the green tea and rooibos extracts further enhance oxidative stress, either through pro-oxidant effects involving auto-oxidation and/or by inducing complex II defects. Oxidative stress resulting from complex II inhibition and auto-oxidation of green tea polyphenols has been associated with induction of apoptosis in different cell lines [62,63,64]. In the present study, rooibos and green tea extracts displayed similar activity by enhancing the UVB-induced reduction in cell viability and this was associated with the induction of apoptosis in the keratinocytes. As pre-neoplastic or initiated cells are more prone to undergo apoptosis due to DNA damage [65], it is likely that the pro-oxidant properties of these extracts, and the resultant oxidative stress, accelerate UVB damaged in the keratinocytes thereby facilitating apoptosis and subsequent removal of inflamed cells containing high levels of biologically active icIL-1α. 

The honeybush species displayed different effects compared to green tea and rooibos. This could be related to the activity of their major polyphenolic constituents, xanthones and flavanones. UVB-irradiated cells exhibited resistance to reduction of cell viability and inhibition of cell proliferation by the methanol extracts of *C. intermedia* and *C. subternata*. This indicated that these honeybush extracts have a cytoprotective effect against UVB-induced damage related to the induction of oxidative stress. The cytoprotective effect was further emphasised by the reduction of UVB-induced caspase-3 activity associated with apoptosis that was exhibited by the methanol extracts of *C. intermedia* and *C. subternata.* Since the methanol extracts have high levels of xanthones and flavanones, the cytoprotective effect of honeybush species may be mediated by these major polyphenolic constituents, as suggested previously [24]. Mangiferin has been shown to protect human skin keratinocytes against oxidative cell death [66] while hesperidin has been found to exhibit protective effects against radiation-induced DNA damage and cell proliferation in bone marrow cells [67]. Other phenolic constituents of honeybush, i.e., the flavanone, eriocitrin, was reported to be more effective at protecting lipid membrane against oxidative stress than hesperidin [68], while the flavone, scolymoside is also known to exhibit a strong radical scavenging activity [69]. Of interest is that a high level of scolymoside associated with the IC_50_ against cell viability of the methanol extract of *C. subternata*, which exhibited a protection against apoptosis. In addition, the protection against these cell survival parameters by the methanol extract of *C. subternata* was also associated with high levels of phloretin-3′,5′-di-*C*-glucoside and iriflophenone-3-*C*-glucoside. Therefore, it is possible that the xanthones may be acting additively and/or synergistically with hesperidin and other polyphenolic constituents to protect against UVB-induced oxidative stress. 

The cytoprotective effect of honeybush has also been reported to be dependent on a specific xanthone-to-flavanone ratio and/or the FLAVA constituents. A respective decrease in the xanthone-to flavanone ratio and increase in the FLAVA content seems to mediate the cytotoxic activity of the extracts [24]. This also became apparent in the current study as an increased activity in the reduction of cell viability, inhibition of cell proliferation and an increased induction of apoptosis was noticed by the aqueous extracts of both honeybush species. Similar to green tea and rooibos, the cytotoxic effect of the honeybush species may be mediated by pro-oxidant polyphenol/iron interactions by these constituents. In this regard the pro-oxidant activity of the tannin-like polymeric compounds, including the proanthocyanidins that occur in higher levels in the aqueous extracts, could play a determining role. However, the role of these polymeric constituents in the pro-oxidant and pro-apoptotic activity of honeybush still needs to be elucidated. With respect to green tea far higher FLAVA levels were also associated with the IC_50_ value for the reduction in cell viability and icIL-1α by the aqueous extract. This would imply that more hydrophilic constituents may exhibited a protective effect against cell death counteracting the effect of the catechins. In contrast, the two rooibos extracts display remarkably similar effects when comparing the levels of the polyphenolic constituents associated with their IC_50_ values for reduction in cell viability and icIL-1α accumulation. In both cases the aqueous extract also exhibited a reduced effect on the induction of apoptosis. Therefore, the relative differences in the anti- and/or pro-oxidant potential are associated with variations in the type of polyphenolic constituents as well as their relative concentrations in comprising the methanol and aqueous extracts of green tea and the herbal teas.

Regarding the modulation of UVB-induced icIL-1α accumulation by the honeybush species, the aqueous extract of *C. intermedia* inhibited icIL-1α production in a dose dependent manner, but this effect was far weaker than that of green tea and rooibos extracts. The inhibitory effect was dose-dependent and correlated strongly with the reduction in cell viability and induction of caspase-3 activity, suggesting that the anti-inflammatory effect follows a similar mechanism involving apoptosis as has been described earlier for green tea and rooibos. On the other hand, the methanol extracts of both honeybush species enhanced icIL-1α accumulation, suggestive of a pro-inflammatory effect that was closely associated with a reduction in UVB-induced apoptosis without adversely affecting cell viability. The pro-inflammatory activity of the honeybush species is possibly mediated by two mechanisms. In the first mechanism, inflamed cells with high levels of biologically active icIL-1α are protected from entering into apoptosis through antioxidant mechanisms that involve polyphenol/iron interactions. In the second mechanism, known/unknown compounds in honeybush may stimulate icIL-1α production through mechanisms that involve transcription modulation, but this needs further investigation. Since the aqueous extract of *C. subternata* enhanced icIL-1α production by reducing cell viability as well as inducing apoptosis, could be related to the second proposed mechanism involving transcription regulation. It would appear that thresholds exist for the effective removal of UVB-induced icIL-1α in keratinocytes by the herbal teas which is governed by different mechanisms involving complex keratinocyte/polyphenolic interactions. 

In summary, the current study indicated that rooibos extracts and the aqueous extract of *C. intermedia*, similar to green tea extracts, exhibit indirect anti-inflammatory effects against UVB-induced damage in keratinocytes by removing damaged inflamed skin cells with high levels of icIL-1α via apoptosis. The underlying mechanisms in this indirect “anti-inflammatory” effect may involve pro-oxidative polyphenol/iron interactions that result from mitochondrial dysfunction and oxidative damage targeting damaged cells to undergo apoptosis. On the other hand, the methanol extracts of honeybush exhibited a pro-inflammatory activity mainly associated with cytoprotective effects, presumably mediated by antioxidant mechanisms that also involve polyphenol/iron protective interactions. However, there might also be a stimulatory role involving transcription modulation in the pro-inflammatory activity of the aqueous extract of *C. subternata*. The indirect anti-inflammatory effect of green tea, rooibos and the aqueous extract of *C. intermedia* or specific combination thereof may be useful in the prevention of UVB-induced chronic inflammation and photo-carcinogenesis. These extracts may be considered as an additive in skin care products directed for use following excessive sun exposure. Although methanol extracts of honeybush seem to protect against UVB-induced harmful effects, these extracts should rather be further characterised in a pre-exposure model for protection against UVB induced oxidative stress. In this regard, mangiferin has been shown to possess cytoprotective and anti-genotoxic activity in vitro [70]. A recent study also reported on the antioxidant and anti-inflammatory potential of fermented *C. intermedia* extract in a pre-treatment UVB-HaCaT model further supporting the proposed photo protective properties of honeybush in the skin [71]. Future studies on rooibos and species-specific honeybush aqueous extracts should investigate the role of iron-related pro-oxidant mechanisms in effecting mitochondrial dysfunction, enhancement of oxidative stress and apoptosis as well as the subsequent removal of UVB damaged keratinocytes and the attenuation of inflammation.

## 4. Materials and Methods

### 4.1. Chemicals

Bovine serum albumin (BSA) was purchased from Sigma-Aldrich (St. Louis, MO, USA), heat inactivated fetal bovine serum (FBS) from Invitrogen (Carlsbad, CA, USA), and RPMI-1640, Dulbecco’s phosphate buffered saline (DPBS), l-glutamine, trypsin-versene and Hank’s buffered salt solution (HBSS) from Lonza, (Braine-l’Alleud, Belgium). Human recombinant IL-1α ELISA kit was purchased from R&D systems, Minneapolis, MN, USA, CytoTox 96®. The non-radioactive cytotoxicity assay, CellTiter-Glo luminescent cell viability, Cell proliferation ELISA and BrDU chemiluninescent were supplied by Roche (Mannheim, Germany), and Caspase-3/7 assay by Promega (Madison, WI, USA). BDH Chemical Ltd. (Poole, England) supplied Triton-x100 and ICN Biomedicals Inc (Santa Ana, CA, USA) supplied Tween®-20.

### 4.2. Plant Material and Extract Preparation

“Unfermented” (unoxidized) rooibos (*Aspalathus linearis*), “unfermented honeybush” (*C. intermedia* and *C. subternata*) and *g*reen tea (*Camellia sinensis*) were used to prepare aqueous and methanol extracts. Extract preparation and characterisation in terms individual polyphenol content by high performance liquid chromatography and global parameters, i.e., total polyphenol (TP) and flavanol/proanthocyanidin (FLAVA) content, using colorimetric assays, have been described previously [24]. The FLAVA content refers to *p*-dimethylaminocinnamaldehyde (DMACA)-reactive substances. In the case of rooibos and honeybush the reaction is attributed to polymeric proanthocyanidin type compounds while in green tea monomeric flavanols are the main constituents.

### 4.3. UVB/Keratinocyte Inflammatory Cell Model

Spontaneously immortalised keratinocytes (HaCaT) were a gift from the Department of Human Biology at the University of Cape Town, South Africa. The cells were cultured in RPMI-1640 supplemented with inactivated fetal bovine serum (10%), l-glutamine (2 mM) and incubated in a humidified atmosphere of 5% CO_2_/95% air at 37 °C. Cells were passaged every 3 days at 70% to 80% confluence in a 1:3 split ratio and passages 70 to 80 were utilised in experiments. When conducting the experiments, cells were seeded (100 µL) in 96-well tissue culture microtitre plates at a density of 3 × 10^4^. After removing the cultured media the cells were overlaid with Dulbecco’s phosphate buffered saline (DPBS; 100 µL) and exposed to UVB light (80 mJ/cm^2^) without the plastic lid. A UVIlink UV Crosslinker (UVitek Ltd., Aberdeen, UK) was fitted with six 8 Watt UV tubes giving a wavelength of 302 nm (Vilber Lourmat, Collégien, France). After UVB exposure, cells were subsequently washed with DPBS (100 µL) and supplemented with cultured medium (100 µL) containing the different treatment regimens as described below and cells were incubated for 24 h. Following incubation, samples were collected and analysed for icIL-1α production, cell viability, cell proliferation and apoptosis. Control plates were not exposed to UVB light.

### 4.4. Modulation of IL-1α Production and Different Cell Growth Parameters

Irradiated or non-irradiated cells were either exposed to fresh RPMI-1640 media containing 0.5% FBS (control wells) or to 0.5% FBS RPMI-1640 containing varying concentrations of the extracts (experimental wells). The extracts were prepared in the culture media using DMSO and filtered (0.22 µm). The different extract concentrations used (mg/mL) were as follows: green tea extracts (MeOH: 0.11–0.007; Aq: 0.43–0.028); rooibos (MeOH: 0.19–0.01; Aq: 0.55–0.03); *C. intermedia* (MeOH: 1.5–0.05; Aq: 0.79–0.05); and *C. subternata* (MeOH: 1.80–0.18; Aq: 1.00–0.09). Final concentration of DMSO in the cultured media for both the control and experimental treatments did not exceed 0.5%. Microtitre plates were incubated for 24 h and the effect on cell viability and cell proliferation monitored as described below. The effect on IL-1α and apoptosis was also monitored, but only in UVB irradiated cells in the presence of varying concentrations of the extracts. Separate experiments were conducted to evaluate the interrelationship between IL-1α and cell viability, as well as between apoptosis and cell viability. Each experiment was repeated five times for each extract concentration and all analyses was repeated two to three times.

### 4.5. Cell Growth Parameters

#### 4.5.1. Cell Viability Assay

The CellTiter-Glo Luminescent kit was used to monitor ATP production in cells cultured using white solid microplates (Porvair Sciences, Fareham, UK). The microplates were first equilibrated at room temperature for 30 min, luciferase agent added to the wells and the plates rotated for 2 min, after which they were incubated at room temperature for 10 min in the dark. ATP content was monitored using the Veritas^TM^ microplate luminometer (Promega, Madison, WI, USA). The luminescent signal was measured in relative light units (RLU) and data expressed as a percentage (%) of the control cells as depicted in Equation (1):

(1) $$\%\;ATP\;production\;=\;RLU_{treated\;cells}\;/\;RLU_{control}\;\times \;100$$

#### 4.5.2. Cell Proliferation Assay

Cell proliferation was determined using the BrdU chemiluminescent immunoassay kit measuring BrdU incorporation during DNA synthesis in cells, plated in black solid microplates (Porvair Sciences), following the manufacturer’s instructions. Briefly, after 24 h incubation with the extracts added, cells were labelled using BrdU solution (10 µL). The microplates were incubated for 2 h at 37 °C during labelling. After incubation, the media were decanted, cells fixed with denaturing solution (200 µL) and incubated at room temperature for 30 min. After removal of the denaturing solution, cells were incubated for 1 h 30 min with the BrdU antibody (100 µL) added. Plates were washed 3 times with saline, treated with the substrate (100 µL), covered with foil and rotated for 3 min before quantification of DNA synthesis using a Veritas^TM^ microplate luminometer (Promega) The luminescent signal was measured in relative light units (RLU) and data expressed as a percentage (%) of the control cells as for ATP production, as depicted in Equation (2):

(2) $$\%\;Cell\;proliferation\;=\;RLU_{treated\;cells}\;/\;RLU_{control}\;\times \;100$$

IC_50_ values for ATP and BrDU assays were calculated on the basis of the best fit for dose-response data using the 4-parameter logistic curve (Sigmoidal variable slope) in GraphPad Prism version 5.04 for Windows (GraphPad Software, La Jolla, CA, USA). 

#### 4.5.3. Modulation of Apoptosis

The ability to enhance apoptosis in cells was determined for extracts of green tea, rooibos and the two flavanone–rich honeybush species (*C. intermedia* and *C. subternata*), selected based on their reactivity against cell viability and proliferation. Assays were conducted in clear microplates and cells lysed in lysis buffer (20 μL) in combination with one freeze–thaw cycle. Cell lysates were transferred (25 µL) into a white solid microplates and incubated in the presence of the caspase 3/7 reagent (25 µL) in the dark at room temperature for 1 h. Analyses were conducted using a Veritas microplate luminometer. The data, generated as relative light units, were expressed as fold increase compared to the control treatment regimens. 

#### 4.5.4. Modulation of IL-1α Accumulation

The concentrations of the different extracts utilised to determine their activity against IL-1α production were selected at sub-cytotoxic levels based on the activity (IC_50_) of the extracts against cell viability (ATP). Intracellular (icIL-1α) and extracellular interleukin-1α (exIL-1α) were determined in cell lysates and supernatants, respectively, using an IL-1α ELISA kit based on the manufacturer’s instruction. Absorbance was measured at 450 nm, using a Dynex plate reader (Dynex Technologies, Chantilly, VA, USA), data were analysed using an IL-1α standard curve generated by GraphPad prism and expressed as pg/mL of the supernatant or cell lysate.

### 4.6. Statistics

All parameters were tested for normality using the Kolmogorov–Smirnov Test, as well as for homogeneity of group variances using Levene’s Test. Group differences for the parametric parameters, as well as those that could be transformed were then tested using One-way ANOVA and post-hoc Tukey Test for multiple pairwise comparisons between the means of all the groups. When only two groups were compared the student T-test was used. For the non-parametric Kruskal–Wallis Test (Rank Sum for >2 groups) was used assess group differences using the post-hoc Tukey-type test for pairwise groups comparisons. Where only two groups were compared, the non-parametric Wilcoxon Two-sample Test was used for comparison. Spearman correlations were used to calculate all the correlation coefficients. Statistical analyses were performed with SAS v9.2 (SAS institute Inc., Cary, NC, USA) and statistical significance was considered at 5% (*p* < 0.05).

## 5. Conclusions

Application of rooibos extracts containing high levels of monomeric flavonoids and polymeric proanthocyanidins following UVB exposure might prevent cancer development in the skin by facilitating the removal of damaged cells with pro-inflammatory activity via stimulation of UVB-induced apoptosis, as indicated in the UVB/HaCaT inflammatory cell model. The phenolic constituents of honeybush extracts may have different cytotoxic and/or cytoprotective roles when compared to rooibos. The aqueous extracts of honeybush, containing higher levels of polymeric tannin-like compounds including the proanthocyanidins, may also exhibit the same pro-apoptotic and indirect anti-inflammatory effects as rooibos extracts following UVB exposure. However, honeybush methanol extracts containing higher levels of the xanthones, mangiferin and iso-mangiferin, and flavanone, hesperidin, may be useful in protecting the skin against UVB-induced oxidative stress due to their cytoprotective effects implying their use in sunscreens prior to UVB exposure. Herbal tea extracts may be useful in controlling skin cancer by: (i) targeting initiation to remove the DNA damaged cell via apoptosis; and (ii) delaying the promotion stage by preventing the onset of inflammation via the induction of apoptosis and selectively inhibiting their growth. However, the levels of the active polyphenolic compounds in extracts as well as their biological properties should be standardised through a set of in vitro antioxidant and cell-based assays that, together with the chemical characterisation, could be used as quality control parameters to ensure the chemopreventive efficacy of the extracts in skin. 

## Figures and Tables

**Table 1:** Comparative effect of green tea and herbal tea extracts on growth parameters of UV irradiated and non-irradiated HaCaT cells.

**Cell Viability (ATP IC_50_ (mg/mL))**					
**Extract**	**UVB Irradiation**	**Green Tea**	**Rooibos**	***C. intermedia***	***C. subternata***
Methanol	(−)	0.08 ± 0.01 ^c^_A_ *	0.15 ± 0.02 ^c^_A_	1.25 ± 0.21 ^b^	1.65 ± 0.14 ^a^
	(+)	0.06 ± 0.01 ^a^_B_ *	0.10 ± 0.01 ^a^_B_ *	>1.46	>1.80
Aqueous	(−)	0.14 ± 0.01 ^c^_A_	0.13 ± 0.01 ^c^_A_	0.49 ± 0 04 ^b^_A_ *	0.89 ± 0.12 ^a^_A_ *
	(+)	0.14 ± 0.02 ^c^_A_	0.13 ± 0.01 ^c^_A_	0.41 ± 0.10 ^b^_A_	0.72 ± 0.11 ^a^_A_
**Cell proliferation (BrdU IC_50_ (mg/mL))**					
Methanol	(−)	0.06 ± 0.02 ^b^_A_	0.06 ± 0.01 ^b^_A_	0.37 ± 0.08 ^a^	0.27 ± 0.03 ^a^
	(+)	0.09 ± 0.01 ^a^_A_	0.08 ± 0.01 ^a^_A_	>0.71	>1.80
Aqueous	(−)	0.07 ± 0.01 ^b^_A_	0.08 ± 0.01 ^b^_A_	0.33 ± 0.07 ^a^_A_	0.31 ± 0.04 ^a^_A_
	(+)	0.10 ± 0.02 ^c^_A_	0.10 ± 0.01 ^c^_A_	0.38 ± 0.05 ^b^_B_	0.53 ± 0.07 ^a^_B_

Values represent mean ± standard deviation of 4–5 replicates and at least 2 independent experiments. Significant differences (*p* < 0.05) between the different extracts are indicated with differing lower case letters in superscript (in a row). Significant differences between non-irradiated and UVB-irradiated cells for each extract type are indicated with differing upper case letters in subscript (in a column). Means followed by the same letter do not differ significantly. * Significant difference (*p* < 0.05) between methanol and aqueous extracts in the absence (−) and presence (+) of UVB irradiation. Abbreviations: IC_50_, concentration (mg/mL) yielding 50% inhibition; ATP, adenosine triphosphate; BrdU, 5-bromo-2′-deoxyuridine; (−), cells not exposed to UVB light; (+), cells exposed to UVB light (80 mJ/cm^2^).

**Table 2:** Modulation of UVB-induced apoptosis and cell viability (ATP production) by methanol and aqueous extracts of green tea and different herbal teas.

Extracts	Unit Measurement	Controls		Methanol Extracts (mg/mL)			Aqueous Extracts (mg/mL)		
Green tea		**(−) UVB**	**(+) UVB**	**0.11**	**0.05**	**0.03**	**0.43**	**0.22**	**0.11**
	*Casp-3 fold increase*	1.00 ± 0.19 ^d^	3.89 ± 0.68 ^c^	8.16 ± 0.74 ^a^	7.82 ± 0.76 ^a^	6.10 ± 0.21 ^b^	6.51 ± 0.77 ^a^	6.54 ± 0.48 ^a^	6.47 ± 0.81 ^a^
	% ATP production	100.00 ± 3.97 ^a^	74.63 ± 4.79 ^b^	27.74 ± 2.35 ^d^	49.16 ± 5.51 ^c^	69.47 ± 5.05 ^b^	8.90 ± 3.08 ^d^	37.98 ± 4.83 ^c^	± 7.36 ^c^
Rooibos				**0.19**	**0.10**	**0.05**	**0.55**	**0.28**	**0.14**
	*Casp-3 fold increase*	1.00 ± 0.14 ^e^	3.59 ± 0.62 ^d^	10.12 ± 0.88 ^a^	9.03 ± 0.66 ^b^	7.44 ± 0.49 ^c^	5.14 ± 0.50 ^a^	4.61 ± 0.36 ^b^	3.56 ± 0.19 ^d^
	% ATP production	100.00 ± 3.97 ^a^	74.63 ± 4.79 ^b^	28.43 ± 3.41 ^d^	48.18 ± 3.32 ^c^	66.74 ± 4.02 ^b^	15.94 ± 2.34 ^d^	29.56 ± 3.36 ^c^	48.94 ± 5.55 ^b^
*C. intermedia*		**(−) UVB**	**(+) UVB**	**0.73**	**0.37**	**0.18**	**0.79**	**0.39**	**0.20**
	*Casp-3 fold increase*	1.00 ± 0.16 ^c^	3.24 ± 0.44 ^a^	1.13 ± 0.16 ^c^	1.98 ± 0.12 ^b^	2.80 ± 0.29 ^a^	5.38 ± 0.47 ^b^	4.76 ± 0.05 ^b^	3.17 ± 0.42 ^a^
	% ATP production	100.00 ± 3.80 ^a^	88.54 ± 8.84 ^b^	69.76 ± 2.92 ^c^	87.27 ± 5.25 ^b^	88.09 ± 3.59 ^b^	34.87 ± 2.39 ^d^	52.70 ± 5.46 ^b^	70.22 ± 6.27 ^c^
*C. subternata*				**0.71**	**0.36**	**0.18**	**0.75**	**0.36**	**0.18**
	*Casp-3 fold increase*	1.00 ± 0.13 ^c^	3.44 ± 0.26 ^a^	2.44 ± 0.26 ^b^	2.87 ± 0.63 ^b^	3.77 ± 0.27 ^a^	5.87 ± 0.46 ^b^	4.50 ± 0.45 ^b^	4.00 ± 0.36 ^a^
	% ATP production	100.00 ± 3.87 ^a^	92.37 ± 3.88 ^b^	85.12 ± 4.03 ^b^	94.50 ± 2.33 ^b^	93.78 ± 4.12 ^b^	56.94 ± 4.29 ^c^	82.76 ± 3.67 ^b^	93.46 ± 4.98 ^b^

Values represent mean ± standard deviation of four to five repetitions and two independent experiments. Statistically significant differences between the different methanol and aqueous extract concentrations were compared separately to the controls. Means followed by the same letter (superscript in a row) do not differ significantly, if letters differ then *p* < 0.05. *Abbreviations*: ATP—adenosine triphosphate; Casp-3—caspase-3; % ATP production calculated against control cells; (−) UVB—cells that were not exposed to ultraviolet-B light; (+) UVB—cells exposed to UVB light (80 mJ/cm^2^).

**Table 3:** Modulation of exIL-1α (pg/mL) in the supernatant by the methanol and aqueous extracts of green tea and rooibos herbal tea.

Extract Type	(−) UVB	(+) UVB	Concentration (mg/mL)			
			0.107	0.054	0.027	0.013
Gr_Methanol	2.57 ± 0.85a	3.62 ± 0.31a	1.35 ± 0.04b	1.53 ± 0.15b	1.84 ± 0.31b	2.27 ± 0.57a
Gr_Aqueous	2.92 ± 0.85a	3.84 ± 0.56a	2.01 ± 0.17b	1.31 ± 0.20b	1.15 ± 0.07b	1.62 ± 0.15b
RB_Methanol	1.62 ± 0.36a	1.71 ± 0.24a	1.43 ± 0.10a	1.49 ± 0.36a	1.62 ± 0.21a	2.21 ± 0.18a
RB_Aqueous	1.62 ± 0.36a	1.71 ± 0.24a	1.31 ± 0.29a	1.26 ± 0.21a	1.67 ± 0.49a	1.20 ± 0.10a

Values represent mean ± standard deviation of 4 to 5 repetitions and two independent experiments. Statistically significant differences were indicated with different lower case letters (in a row) compared to the controls (*p* < 0.05). *Abbreviations*: (−) UVB—cells that were not exposed to ultraviolet-B light; (+) UVB—cells exposed to UVB light (80 mJ/cm^2^; exIL-1α—extracellular interleukin-1α; Rg—green tea; RB—rooibos herbal tea.

**Table 4:** Modulation of IL-1α and accumulation by the different honeybush extracts in relation to their effect on cell viability (ATP content).

Parameter	Controls		Methanol Extracts (mg/mL)				Aqueous Extracts (mg/mL)			
			***C. intermedia***							
	(−) UVB	(+) UVB	0.73	0.37	0.18	0.09	0.79	0.39	0.20	0.10
icIL-1α (pg/mL)	8.35 ± 1.0 ^d^	28.14 ± 2.41 ^b^	40.88 ± 2.78 ^a^	30.77 ± 2.34 ^b^	23.4 ± 2.52 ^b^	22.6 ± 2.24 ^b^	17.93 ± 7.45 ^c^	22.11 ± 7.35 ^b^	28.95 ± 7.43 ^b^	29.52 ± 7.63 ^b^
exIL-1α (pg/mL)	nd	1.41 ± 0.20 ^b^	1.73 ± 0.21 ^b^	1.75 ± 0.43 ^b^	1.93 ± 0.73 ^b^	2.42 ± 0.40 ^a^	2.63 ± 0.11 ^a^	1.39 ± 0.25 ^b^	1.35 ± 0.12 ^b^	1.46 ± 0.10 ^b^
% ATP production	100.0 ± 5.0 ^a^	83.96 ± 3.65 ^b^	82.29 ± 7.43 ^b^	88.63 ± 5.90 ^b^	81.91 ± 5.07 ^b^	84.31 ± 2.79 ^b^	35.15 ± 7.13 ^e^	55.64 ± 8.02 ^d^	68.25 ± 7.62 ^c^	74.82 ± 10.54 ^b^
			***C. subternata***							
	(−) UVB	(+) UVB	0.71	0.36	0.18	0.09	0.75	0.36	0.19	0.09
icIL-1α (pg/mL)	15.53 ± 3.51 ^d^	36.10 ± 5.73 ^b^	51.94 ± 4.2 ^a^	43.55 ± 9.16 ^b^	40.83 ± 8.24 ^b^	36.23 ± 7.87 ^b^	56.53 ± 4.14 ^a^	45.32 ± 3.97 ^c^	48.02 ± 5.63 ^c^	39.91 ± 9.92 ^bc^
exIL-1α (pg/mL)	2.72 ± 0.71 ^b^	3.72 ± 0.22 ^a^	2.40 ± 0.19 ^b^	2.62 ± 0.42 ^b^	2.65 ± 0.19 ^b^	2.72 ± 0.28 ^b^	1.22 ± 0.07 ^c^	1.31 ± 0.13 ^c^	1.84 ± 0.20 ^b^	1.74 ± 0.21 ^b^
% ATP production	100.00 ± 4.12 ^a^	89.60 ± 4.41 ^b^	82.07 ± 3.02 ^c^	86.28 ± 2.54 ^b^	88.01 ± 7.66 ^b^	93.48 ± 5.92 ^b^	42.02 ± 6.94 ^e^	62.22 ± 7.00 ^d^	77.33 ± 9.51 ^c^	79.37 ± 8.52 ^c^

Values represent mean ± standard deviation of four to five repetitions and two independent experiments. Statistically significant differences were indicated with different lower case letters in superscript (in a row) for each extract separately compared to the controls (*p* < 0.05). *Abbreviations*: (−) UVB—cells that were not exposed to ultraviolet-B light; (+) UVB—cells exposed to UVB light (80 mJ/cm^2^); icIL-1α—intracellular interleukin-1α; exIL-1α—extracellular interleukin-1α; % ATP production—indicate the percentage ATP production calculated from the relevant control cells; ATP—adenosine triphosphate; nd—not detected.

**Table 5:** Correlation among cell viability, caspase-3 activity (fold) and icIL1-α content as affected by green tea and herbal tea extracts.

Extract	icIL-1α_ATP	Casp-3_F_ATP	Casp-3_F _icIL-1α
**Green Tea**			
Methanol	0.618 (<0.0001)	−0.895 (<0.0001)	−0.863 (<0.0001)
Aqueous	0.754 (<0.0001)	−0.880 (<0.0001)	−0.833 (<0.0001)
**Rooibos**			
Methanol	0.742 (<0.0001)	−0.878 (<0.0001)	−0.899 (<0.0001)
Aqueous	0.796 (<0.0001)	−0.848 (<0.0001)	−0.876 (<0.0001)
***C. intermedia***			
Methanol			−0.494 (0.0005)
Aqueous	0.720 (<0.0001)	−0.820 (<0.0001)	−0.555 (<0.0001)
***C. subternata***			
Methanol	−0.309 (0.0017)	−0.597 (<0.0001)	
Aqueous	−0.229 (0.023)	−0.822 (<0.0001)	0.517 (0.0001)

Values represent correlation coefficients (r) with p values indicated in brackets. *Abbreviations*: Casp-3_ F—caspase-3 fold increase; icIL-1α_ATP—correlation between intracellular icIL-1α inhibition and ATP inhibition; Casp-3_F_ATP—correlation between caspase-3 fold increase and ATP inhibition; Casp-3_F_icIL-1α—correlation between caspase-3 fold increase and icIL-1α inhibition; ATP—adenosine triphosphate.

**Table 6:** Polyphenol concentration (µg/mL) of green tea and rooibos extracts expressed as a function of the IC_50_ values for the inhibition of icIL-1α accumulation and reduction of cell viability following UVB irradiation in HaCaT keratinocytes.

	Polyphenols **	icIL_1α		Cell Viability (% ATP)	
		Methanol	Aqueous	Methanol	Aqueous
Green tea		0.08 mg/mL *	0.28 mg/mL *	0.06 mg/mL *	0.14 mg/mL *
	TP	28.06 ± 2.62 a	41.86 ± 5.62 b	15.03 ± 0.99 a	35.07 ± 2.30 b
	FLAVA	10.58 ± 0.30 a	20.18 ± 0.81 b	7.94 ± 0.22 a	10.86 ± 043 b
	EGCG	8.95 ± 0.24 a	29.10 ± 0.39 b	6.72 ± 0.18 a	6.45 ± 0.21 a
	ECG	1.63 ± 0.03 a	1.94 ± 0.11 b	1.22 ± 0.02 a	1.05 ± 0.06 a
	EGC	3.38 ± 0.14 a	8.932 ± 0.83 b	2.54 ± 0.11 a	4.48 ± 0.45 b
	EC	1.19 ± 0.08 a	2.93 ± 0.30 b	0.90 ± 0.06 a	1.58 ± 0.16 b
	Catechin	0.11 ± 0.01 a	0.29 ± 0.01 b	0.08 ± 0.01 a	0.16 ± 0.01 b
	**Total flavanols**	**15.26 ± 0.50 a**	**25.47 ± 0.73 b**	**11.45 ± 0.37 a**	**13.72 ± 0.39 b**
Rooibos		0.14 mg/mL *	0.21 mg/mL *	0.10 mg/mL *	0.13 mg/mL *
	TP	49.11 ± 4.82 a	52.62 ± 5.97 a	35.08 ± 3.44 a	32.57 ± 3.70 a
	FLAVA	3.26 ± 0.20 a	3.24 ± 0.27 a	2.71 ± 0.16 a	2.34 ± 0.20 a
	Aspalathin	17.39 ± 0.20 a	17.61 ± 0.44 a	12.44 ± 0.14 a	10.90 ± 0.27 b
	Nothofagin	3.86 ± 0.05 a	3.50 ± 0.06 b	2.76 ± 0.04 a	2.17 ± 0.04 b
	**Total DHC**	**21.25 ± 0.23 a**	**21.12 ± 0.38 a**	**15.18 ± 0.17 a**	**13.07 ± 0.24 b**
	Iso-orientin	2.21 ± 0.01 a	2.30 ± 0.41 a	1.58 ± 0.01 a	1.42 ± 0.25 a
	Orientin	1.62 ± 0.01 a	1.86 ± 0.30 a	1.16 ± 0.01 a	1.15 ± 0.19 a
	Vitexin	0.22 ± 0.0 a	0.25 ± 0.0 a	0.16 ± 0.0 a	0.16 ± 0.0 a
	Isovitexin	0.32 ± 0.0 a	0.32 ±0.03 a	0.26 ± 0.0 a	0.20 ± 0.02 a
	L7Glc	0.25 ± 0.13 a	0.09 ± 0.02 a	0.18 ± 0.01 a	0.06 ± 0.01 b
	**Total flavones**	**4.67 ± 0.01 a**	**4.83 ± 0.62 a**	**3.34 ± 0.01 a**	**2.99 ± 0.45 a**
	Rutin	0.60 ± 0.0 a	0.76 ± 0.0 b	0.43 ± 0.0 a	0.47 ± 0.0 a
	Hyperoside	0.49 ± 0.01 a	0.31 ± 0.16 a	0.35 ± 0.01 a	0.19 ± 0.10 b
	Isoquercitrin	0.63 ± 0.0 a	0.42 ± 0.21 a	0.45 ± 0.0 a	0.26 ± 0.12 b
	QROB	1.62 ± 0.0 a	1.58 ± 0.0 a	1.16 ± 0.01 a	0.98 ± 0.12 a
	**Total flavanols**	**3.35 ± 0.01 a**	**3.06 ± 0.35 a**	**2.39 ± 0.01 a**	**1.89 ± 0.22 b**
	PPAG	0.54 ± 0.03 a	0.89 ± 0.02 b	0.39 ± 0.02 a	0.55 ± 0.01 b

Values represent means ± standard deviations of triplcate deteminations. * IC_50_ values as depicted in Table 1 (Cell viability) and Figure 1 (icIL-1α). ** Data calculated using the individual polyphenol content of the extracts as previously reported [24]. Statistically significant (*p* < 0.05) differences were indicated with different lower case letters (in a row) *Abbreviations*: TP—total polyphenols; FLAVA—polymeric tannin-like compounds including proanthocyanidins; IC_50_—concentration yielding 50% inhibition of ATP and icIL-1α; icIL-1α- intracellular interleukin-1α; ATP—adenosine triphosphate; EGCG—epigallocatechin gallate; EGC—epigallocatechin; ECG—epicatechin gallate; EC—picatechin; DHC—dihydrochalchones; L7Glc—luteolin-7-*O*-glucoside; QROB—quercetin-3-*O*-robinobioside; PPAG—phenylpyruvic acid glucoside (Precursor of phenolic compounds).

**Table 7:** Polyphenol concentration (µg/mL) of the aqueous extracts of C. intermedia and C. subternata expressed as a function of the IC_50_ values for the reduction of cell viability and icIL-1α following UVB irradiation.

Polyphenols **		*C. intermedia*		*C. subternata #*
		IC_50_ % ATP (0.41 mg/mL) *	IC_50_ icIL-1α (0.61 mg/mL) *	IC_50_ % ATP (0.72 mg/mL) *
TP		67.45 ± 4.63 a	133.25 ± 10.86	126.00 ± 17.35 b
FLAVA		7.30 ± 0.37 a	6.89 ± 0.55	16.49 ± 0.65 b
Xanthones	Mangiferin	16.30 ± 0.16 a	24.26 ± 0.25	15.98 ± 2.15 a
	Isomangiferin	5.85 ± 0.24 a	8.70 ± 0.24	6.26 ± 1.05 a
	**Total**	**22.16 ± 0.31 a**	**32.96 ± 0.46**	**22.10 ± 3.20 a**
Flavanones	Eriocitrin	0.51 ± 0.02 a	0.76 ± 0.04	2.35 ± 0.18 b
	Hesperidin	3.00 ± 0.23 a	4.47 ± 0.34	5.75 ± 0.15 b
	Eriodictyol-glucoside			2.79 ± 0.14
	**Total**	**3.51 ± 0.25 a**	**5.23 ± 0.38**	**10.89 ± 0.17 b**
Flavones	Luteolin	0.09 ± 0.01 a	0.14 ± 0.01	0.09 ± 0.04 a
	Scolymoside			2.90 ± 0.09
	**Total**	**0.09 ± 0.01 a**	**0.14 ± 0.01**	**3.00 ± 0.06 b**
DHC	Phloretin-3′,5′-di-*C*-glucoside	0.28 ± 0.01 a	0.41 ± 0.01	9.02 ± 1.02 b
Benzophenone	Iriflophenone-3-*C*-glucoside	2.18 ± 0.05 a	2.21 ± 0.07	6.71 ± 0.11 b

Values represent means ± standard deviation of triplicate determinations. # Lacking IC_50_ value for icIL-1α. * IC_50_ values as derived from Table 1 and Table 4. Significant differences (*p* < 0.05) between the means (IC_50_ ATP) in a row) were indicated with different lower case letters. ** Data calculated using the individual polyphenol content of the extracts as previously reported [24]. *Abbreviations*: TP—total polyphenols; FLAVA—flavanols/proanthocyanidins; IC_50_—concentration yielding 50% inhibition.

## References

[1] De Gruijl F.R. (1999). Skin cancer and solar UV radiation. Eur. J. Cancer.

[2] Halliday G.M. (2005). Inflammation, gene mutation and photoimmunosuppression in response to UVR-induced oxidative damage contributes to photocarcinogenesis. Mutat. Res..

[3] Nishisgori C. (2015). Current concept of photocarcinogenesis. Photochem. Photobiol. Sci..

[4] Soehnge H., Outiht A., Ananthaswamy H.N. (1997). Mechanisms of induction of skin cancer induction by UV radiation. Front. Biosci..

[5] Melnikova V.O., Ananthaswamy H.N. (2005). Cellular and molecular events leading to the development of skin cancer. Mutat. Res..

[6] Kim R.H., Armstrong A.W. (2012). Nonmelanoma skin cancer. Dermatol. Clin..

[7] Boukamp P. (2005). Non-melanoma skin cancer: What drives tumor development and progression?. Carcinogenesis..

[8] Chen A.C., Halliday G.M., Damian D.L. (2013). Non-melanoma skin cancer: Carcinogenesis and chemoprevention. Pathology.

[9] Clydesdale G.J., Dandie G.W., Muller H.K. (2001). Ultraviolet light induced injury: Immunological and inflammatory effects. Immunol. Cell Biol..

[10] De Gruijl F.R., van kranen H.J., Mullenders L.H. (2001). UV-induced DNA damage, repair, mutations and oncogenic pathways in skin cancer. J. Photochem. Photobiol. B.

[11] Kim Y., He Y.Y. (2014). Ultraviolet radiation-induced non-melanoma skin cancer: Regulation of DNA damage repair and inflammation. Genes Dis..

[12] Schwarz T. (2005). Mechanisms of UV-induced immunosuppression. Keio. J. Med..

[13] Dinarello C.A. (1997). Interleukin-1. Cytokine Growth Factor Rev..

[14] Stamatas G.N., Morello A.P., Mays D.A. (2013). Early inflammatory processes in the skin. Curr. Mol. Med..

[15] Rider P., Carmi Y., Voronov E., Apte R.N. (2013). Interleukin-1α. Semin. Immunol..

[16] Dinarello C.A. (2011). Interleukin-1 in the pathogenesis and treatment of inflammatory diseases. Blood.

[17] Di Paolo N.C., Shayakhmetov D.M. (2016). Interleukin 1α and the inflammatory process. Nat. Immunol..

[18] Lee W.Y., Locniskar M.F., Fischer S.M. (1994). Interleukin-1α mediates phorbol ester induced inflammation and epidermal hyperplasia. FASEB J..

[19] Rauschmayr T., Nakamura K., Sarkar S., Williams I.R., Kupper T.S. (1996). Inflammatory and hyperproliferative skin disease in mice that express elevated levels of the IL-1 receptor (type I) on epidermal keratinocytes. Evidence that IL-1-inducible secondary cytokines produced by keratinocytesin vivo can cause skin disease. J. Clin. Invest..

[20] Li X., Eckard J., Shah R., Malluck C., Frenkel K. (2002). Interleukin-1α up-regulationin vivo by a potent carcinogen 7,12-dimethylbenz(a)anthracene (DMBA) and control of DMBA-induced inflammatory responses. Cancer Res..

[21] Lee W.Y., Butler A.P., Locniskar M.F., Fischer S.M. (1994). Signal transduction pathway(s) involved in phorbol ester and autocrine induction of interleukin-1 alpha mRNA in murine keratinocytes. J. Biol. Chem..

[22] Hobbs R.M., Watt F.M. (2003). Regulation of interleukin-1α expression by intergrins and epidermal growth factor receptor in keratinocytes from a mouse model of inflammatory skin disease. J. Biol. Chem..

[23] Cohen I., Rider P., Carmi Y., Braiman A., Dotan S., White M.R., Voronov E., Martin M.U., Dinarello C.A., Apte R.N. (2010). Differential release of chromatin-bound IL-1α discriminates between necrotic and apoptotic cell death by the ability to induce sterile inflammation. PNAS.

[24] Magcwebeba T.U., Riedel S., Swanevelder S., Swart P., de Beer D., Joubert E., Gelderblom W.C.A. (2016). The potential role of polyphenols in the modulation of skin cell viability by *Aspalathus linearis* and *Cyclopia* spp. herbal tea extracts in vitro. J. Pharm. Pharmacol..

[25] Cohen C., Dossou G., Rougier A., Roguet R. (1991). Measurement of inflammatory mediators produced by human keratinocytes in vitro: A predictive assessment of cutaneous irritation. Toxicol. In Vitro.

[26] Vazquez R., Nelson M.R., Guzman J.J., Corun C.M., Steinberg M. (2004). Immortalised human keratinocytes: A model system to study the efficacy of therapeutic drugs in response to the chemical warfare agent sulphur mustard. Electron. J. Biotechnol..

[27] Tebbe B., Wu S., Geilen C.C., Eberle J., Kodelja V., Orfanos C.E. (1997). L-ascorbic acid inhibits UVA-induced lipid peroxidation and secretion of IL-1alpha and IL-6 in cultured human keratinocytes in vitro. J. Investig. Dermatol..

[28] Pastore S., Lulli D., Potapovich A.I., Fidanza P., Kostyuk V.A., Dellambra E., de Luca C., Maurelli R., Korkina L.G. (2011). Differential modulation of stress-inflammation responses by plant polyphenols in cultured normal human keratinocytes and immortalized HaCaT cells. J. Dermatol. Sci..

[29] Kostyuk V., Potapovich A., de Luca C. (2010). The promise of plant polyphenols as the golden standard skin anti-inflammatory agents. Curr. Drug Metab..

[30] Mantena S.K., Katiyar S.K. (2006). Grapeseed proanthocyanidins inhibit UV-radiation induced oxidative stress and activation of MAPK and NF-κB signalling in human keratinocytes. Free Rad. Biol. Med..

[31] Potapovich A.I., Lulli D., Fidanza P., Kostyuk V.A., de Luca C., Pastore S., Korkina L.G. (2011). Plant polyphenols differentially modulate inflammatory responses of human keratinocytes by interfering with activation of transcription factors NFκB and AhR and EGFR-ERK pathway. Toxicol. Appl. Pharmacol..

[32] Ramachandran S., Prasad N.R. (2012). Sesamol modulates UVB induced apoptotic and inflammatory signalling in human skin dermal fibroblasts. Int. J. Nutr. Pharmacol. Neurol. Dis..

[33] Katiyar S.K., Rupp C.O., Korman N.J., Agarwal R., Mukhtar H. (1995). Inhibition of 12-O-tetradecanoylphorbol-13-acetate and other skin tumor-promoter-caused induction of epidermal interleukin-1 mRNA and protein expression in SENCAR mice by green tea polyphenol. J. Investig. Dermatol..

[34] Katiyar S.K., Mukhtar H. (1997). Inhibition of phorbol ester tumor-promoter induced 12-*O*-tetradecanoylphorbol-13-acetate caused inflammatory response in SENCAR mouse skin by black tea polyphenols. Carcinogenesis.

[35] Joubert E., Gelderblom W.C., Louw A., de Beer D. (2008). South African herbal teas: *Aspalathus linearis*, *Cyclopia* spp. and *Athrixia phylicoide*—A review. J. Ethnopharmacol..

[36] Joubert E., de Beer D. (2011). Rooibos (*Aspalathus linearis*) beyond the farm gate: From herbal tea to potential phytopharmaceutical. S. Afr. J. Bot..

[37] Joubert E., Joubert M.E., Bester C., de Beer D., de Lange J.H. (2011). Honeybush (*Cyclopia* spp.): From local cottage industry to global markets—The catalytic and supporting role of research. S. Afr. J. Bot..

[38] Marnewick J., Joubert E., Joseph S., Swanevelder S., Swart P., Gelderblom W. (2005). Inhibition of tumour promotion in mouse skin by extracts of rooibos (*Aspalathus linearis*) and honeybush (*Cyclopia intermedia*), unique South African herbal teas. Cancer Lett..

[39] Petrova A., Davids L.M., Rautenbach F., Marnewick J.L. (2011). Photoprotection by honeybush extracts, hesperidin and mangiferin against UVB-induced skin damage in SKH-1 mice. J. Photochem. Photobiol. B.

[40] Na H.K., Mossando K.S., Lee J.Y., Surh Y.I. (2004). Inhibition of phorbol ester induced COX-2 expression by some edible African plants. Biofactors.

[41] Kupper T.S., Chua A.O., Flood P., McGuire J., Gubler U. (1987). Interleukin 1 gene expression in cultured human keratinocytes is augmented by ultraviolet irradiation. J. Clin. Investig..

[42] Freedberg I.M., Tomic-Canic M., Komine M., Blumenberg M. (2001). Keratins and the keratinocytes activation cycle. J. Investig. Dermatol..

[43] Magcwebeba T., Riedel S., Swanevelder S., Bouic P., Swart P., Gelderblom W. (2012). Interleukin-1α induction in human keratinocytes (HaCaT): An in vitro model for chemoprevention in skin. J. Skin Cancer.

[44] Gniadecki R., Thorn T., Vicanova J., Petersen A., Wulf H.C. (2001). Role of mitochondria in ultraviolet-induced oxidative stress. J. Cell. Biochem..

[45] Fresco P., Borges F., Marques M.P.M., Diniz C. (2010). The anticancer properties of dietary polyphenols and its relation with apoptosis. Curr. Pharm. Des..

[46] Vicentini F.T., He T., Shao Y., Fonseca M.J., Verri W.A., Fisher G.J., Xu Y. (2011). Quercetin inhibits UV irradiation-induced inflammatory cytokine production in primary human keratinocytes by suppressing the NF-κB pathway. J. Dermatol. Sci..

[47] Leiro J., Arranz J.A., Yanez M., Ubeira F.M., Sanmartin M.L., Orallo F. (2004). Expression profiles of genes involved in mouse nuclear factor-kappa B signal transduction pathway are modulated by mangiferin. Int. J. Immunopharmacol..

[48] Garrido G., Delgado R., Lemus Y., Rodríguez J., García D., Núñez-Sellés A.J. (2004). Protection against septic shock and suppression of tumor necrosis factor alpha and nitric oxide production on macrophages and microglia by a standard aqueous extract of *Mangifera indica* L. (VIMANG®) Role of mangiferin isolated from the extract. Pharmacol. Res..

[49] Yeh C.-C., Kao S.-J., Lin C.-C., Wang S.-D., Liu C.-J., Kao S.-T. (2007). The immunomodulation of endotoxin-induced acute lung injury by hesperidinin vivo and in vitro. Life Sci..

[50] OyetakinWhite P.O., Tribout H., Baron E. (2012). Protective mechanisms of green tea polyphenols in skin. Oxid. Med. Cell. Longev..

[51] Lu Y.-P., Lou Y.-R., Xie J.-G., Peng Q.-Y., Liao J., Yang C.S., Huang M.-T., Conney A.H. (2002). Topical applications of caffeine or (−)-epigallocatechin gallate (EGCG) inhibit carcinogenesis and selectively increase apoptosis in UVB-induced skin tumors in mice. Proc. Natl. Acad. Sci. USA.

[52] Lu Y.-P., Lou Y.-R., Peng Q.-Y., Xie J.-G., Huang M.-T., Conney A.H. (2004). Stimulatory effect of topical application of caffeine on UVB-induced apoptosis in the epidermis of p53 and Bax knockout mice. Cancer Res..

[53] Han W., Ming M., He Y.-Y. (2011). Caffeine promotes Ultraviolet B-induced apoptosis in human keratinocytes without complete DNA repair. J. Biol. Chem..

[54] Olson E.R., Melton T., Dong Z., Bowden G.T. (2008). Stabilization of quercetin paradoxically reduces its proapoptotic effect on UVB-irradiated human keratinocytes. Cancer Prev. Res..

[55] Perk A.A., Shatynska-Mytsyk A., Gerçek Y.C., Boztaş K., Yazgan M., Fayyaz S., Farooqi A.A. (2014). Rutin mediated targeting of signaling machinery in cancer cells. Cancer Cell Int..

[56] Lee C.-Y., Chien Y.S., Chiu T.H., Huang W.W., Lu C.C., Chiang J.H., Yang J.S. (2012). Apoptosis triggered by vitexin in U937 human leukemia cells via a mitochondrial signalling pathway. Oncol. Rep..

[57] Yuan L., Wei S., Wang J., Liu X. (2014). Iso-orientin induces apoptosis and autophagy simultaneously by reactive oxygen species (ROS)-related p53, PI3K/Akt, JNK, and p38 Signaling pathways in HepG2 cancer cells. J. Agric. Food Chem..

[58] Brenneisen P., Wenk J., Klotz L.O., Wlaschek M., Briviba K., Krieg T., Sies H., Scharffetter-Kochanek K. (1998). Central role of ferrous/ferric iron in the ultraviolet B irradiation-mediated signaling pathway leading to increased interstitial collagenase (matrix-degrading metalloprotease (MMP)-1) and stromelysin-1 (MMP-3) mRNA levels in cultured human dermal fibroblasts. J. Biol. Chem..

[59] Joubert E., Winterton P., Britz T.J., Gelderblom W.C. (2005). Antioxidant and pro-oxidant activities of aqueous extracts and crude polyphenolic fractions of rooibos (*Aspalathus linearis*). J. Agric. Food Chem..

[60] Yoon Y.-S., Byun H.-O., Cho H., Kim B.-K., Yoon G.Y. (2003). Complex II defect via down-regulation of iron-sulfur subunit induces mitochondrial dysfunction and cell cycle delay in iron chelation-induced senescence-associated growth arrest. J. Biol. Chem..

[61] Byun H.O., Kim H.Y., Lim J.J., Seo Y.H., Yoon G. (2008). Mitochondrial dysfunction by complex II inhibition delays overall cell cycle progression via reactive oxygen species production. J. Cell. Biochem..

[62] Yang G.Y., Liao J., Li C., Chung J., Yurkow E.J., Ho C.T., Yang C.S. (2000). Effect of black and green tea polyphenols on c-jun phosphorylation and H_2_O_2_ production in transformed and non-transformed human bronchial cell lines: Possible mechanisms of cell growth inhibition and apoptosis induction. Carcinogenesis.

[63] Elbling L., Weiss R.M., Teufelhofer O., Uhl M., Knasmueller S., Schulte-Hermann R., Berger W., Micksche M. (2005). Green tea extract and (−)-epigallocatechin-3-gallate, the major tea catechin, exert oxidant but lack antioxidant activities. FASEB J..

[64] Lemarie A., Huc L., Pazarentzos E., Mahul-Mellier A.L., Grimm S. (2011). Specific disintegration of complex II succinate:ubiquinone oxidoreductase links pH changes to oxidative stress for apoptosis induction. Cell Death Differ..

[65] Schulte-Herman R., Bursch W., Kraupp-Grasl B., Oberhammer F., Wagner A., Jirtle R. (1993). Cell proliferation and apoptosis in normal liver and preneoplastic foci. Environ. Health Perspect..

[66] Chae S., Piao M.J., Kang A.K., Zhang R., Kim K.C., Youn U.J., Nam K.-W., Lee J.H., Hyun J.W. (2011). Inhibition of matrix metalloproteinase-1 induced oxidative stress from *Anemerhena asphodeloides*. Biosci. Biotechnol. Biochem..

[67] Hosseinimehr S.J., Nemati A. (2006). Radioprotective effects of hesperidin against gamma irradiation in mouse bone marrow cells. Br. J. Radiol..

[68] Joubert E., Richards E.S., Merwe J.D., de Beer D., Manley M., Gelderblom W.C. (2008). Effect of species variation and processing on phenolic composition and in vitro antioxidant activity of aqueous extracts of *Cyclopia* spp. (honeybush tea). J. Agric. Food Chem..

[69] Kim N.M., Kim J., Chung H.Y., Choi J.S. (2000). Isolation of luteolin 7-*O*-rutinoside and esculetin with potential antioxidant activity from the aerial parts of *Artemisia montana*. Arch. Pharm. Res..

[70] Rao B.S., Sreedevi M.V., Nageshwar Rao B. (2009). Cytoprotective and antigenotoxic potential of mangiferin, a glucosylxanthone against cadmium chloride induced toxicity in HepG2 cells. Food Chem. Toxicol..

[71] Im A.-R., Yeon S.H., Lee J.S., Um K.A., Ahn Y.-J., Chae S. (2016). Protective effect of fermented *Cyclopia intermedia* against UVB-induced damage in HaCaT human keratinocytes. BMC Complenment. Altern. Med..

